# Quality in the provision of headache care. 2: defining quality and its indicators

**DOI:** 10.1007/s10194-012-0465-2

**Published:** 2012-06-26

**Authors:** Michele Peters, Crispin Jenkinson, Suraj Perera, Elizabeth Loder, Rigmor Jensen, Zaza Katsarava, Raquel Gil Gouveia, Susan Broner, Timothy Steiner

**Affiliations:** 1 Department of Public Health, University of Oxford, Old Road Campus, Oxford, UK; 2 Ministry of Health Care and Nutrition, Colombo, Sri Lanka; 3 Division of Headache and Pain, Department of Neurology, Brigham and Women’s/Faulkner Hospitals, Boston, MA USA; 4 Department of Neurology, Danish Headache Center, Glostrup Hospital, Glostrup, Denmark; 5 Evangelic Hospital, Unna, Germany; 6 Department of Neurology, Hospital da Luz, Lisbon, Portugal; 7 Manhattan Headache Center, New York, NY USA; 8 Department of Neuroscience, Norwegian University of Science and Technology, Trondheim, Norway; 9 Department of Neuroscience, Imperial College London, London, UK; 10 University of Duisburg-Essen, Essen, Germany

**Keywords:** Quality of care, Definition, Indicators, Headache disorders, Global Campaign against Headache

## Abstract

The objective of this study was to define “quality” of headache care, and develop indicators that are applicable in different settings and cultures and to all types of headache. No definition of quality of headache care has been formulated. Two sets of quality indicators, proposed in the US and UK, are limited to their localities and/or specific to migraine and their development received no input from people with headache. We first undertook a literature review. Then we conducted a series of focus-group consultations with key stakeholders (doctors, nurses and patients) in headache care. From the findings we proposed a large number of putative quality indicators, and refined these and reduced their number in consultations with larger international groups of stakeholder representatives. We formulated a definition of quality from the quality indicators. Five main themes were identified: (1) headache services; (2) health professionals; (3) patients; (4) financial resources; (5) political agenda and legislation. An initial list of 160 putative quality indicators in 14 domains was reduced to 30 indicators in 9 domains. These gave rise to the following multidimensional definition of *quality of headache care*: “Good-quality headache care achieves accurate diagnosis and individualized management, has appropriate referral pathways, educates patients about their headaches and their management, is convenient and comfortable, satisfies patients, is efficient and equitable, assesses outcomes and is safe.” Quality in headache care is multidimensional and resides in nine essential domains that are of equal importance. The indicators are currently being tested for feasibility of use in clinical settings.

## Introduction

Headache disorders are a major cause of public ill-health worldwide [1], generating high needs for health care [2]. These needs are poorly met: headache disorders are under-recognised, under-diagnosed and under-treated [3, 4], so that headache-attributed burdens that could and ought to be alleviated persist at high levels everywhere [5]. This public-health challenge, similar in all countries, gave rise to the Global Campaign against Headache [3, 6], conducted by the UK-registered charitable nongovernmental organization *Lifting The Burden* in official relations with the World Health Organization (WHO) [7].

The ultimate purpose of the Global Campaign is to implement health-care services for headache, appropriate to local systems, resources and needs, that will have the effect of reducing the burden of headache [6]. In putting its mind to this objective, and its achievement, *Lifting The Burden* asked two questions: “What makes headache services good?” and “How is it known whether a particular headache service is good, or needs to be improved?”

In reality, defining quality of care is less easy than it might seem. It is recognized that different definitions of quality are both possible and legitimate, and quality is made up of multiple elements [8]. Donabedian’s view [8]—that quality of care is described in terms of “structure” (the attributes of the settings in which care occurs), “process” (the giving and receiving of care), and “outcome” (the effects that care has on health status)—is widely accepted but may not, on its own, be a complete account of quality. Donabedian suggested seven *pillars* on which quality rests: efficacy, effectiveness, efficiency, optimality, acceptability, legitimacy and equity [9]. These might, collectively, define quality. The Institute of Medicine (IOM) specified six *domains* of quality: safety, timeliness, effectiveness, efficiency, equity and patient/family-centredness [10]. The IOM also offered a definition of quality: “The degree to which health care services for individuals and populations increase the likelihood of desired health outcomes and are consistent with current professional knowledge” [10].

**Fig. 1:**
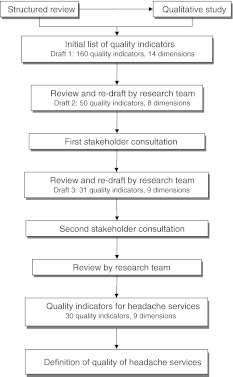
Process of development of a definition of quality and of quality indicators for headache services

Definitions may be generic or disaggregated [11]. Generic definitions are more difficult to operationalize, and they trade specificity for generalizability; disaggregated definitions recognize that quality has different essential components, each providing only a partial picture of quality, but together the components create a more specific description of quality for particular aspects of health care. Campbell and colleagues considered the IOM definition as generic, whereas Donabedian’s fits their view of a disaggregated definition. The two approaches may be at opposite ends of a continuum, but are not inherently incompatible. Nevertheless, it is not apparent that the IOM’s definition, focused as it is on outcomes and professionals, encompasses the six domains in which the IOM believes quality to reside.

These are definitions of quality of care applied to services generally, and it is therefore unlikely that they can cater for the particular requirements of specific conditions. Disease-specific definitions of quality are needed, but, for headache, none exists. Two initiatives, one in the United States of America (US) [12] and one in the United Kingdom (UK) [13], sought to develop quality indicators for headache care but, in both, headache was only one of a large number of conditions for which this was attempted. The indicators were entirely clinical: the 21 US indicators covered three main domains—symptoms, examinations and medications—and the 11 UK indicators covered diagnosis, referral and treatment. Within headache, the US indicators were developed for migraine only, compromising their applicability to other recurrent headache disorders. Both sets of indicators were formulated by a panel of experts, including headache specialists, experts in quality measurement and managed care, and health-care purchasers. Input from headache patients was not sought in either study, even though values placed by patients on the different dimensions of quality are likely to differ from those of health professionals and managers [14]. Furthermore, in each case the indicators were created for one locality (US or UK), whilst what is appropriate in one may not be so in another [15]: not only do perceptions of quality depend upon culture, past experience and expectation but what is reasonably achievable in terms of quality varies with health-service resources and infrastructure. Indicators cannot be transferred between countries without consideration of these factors [15].

In short, *Lifting The Burden* had neither a definition nor a set of quality indicators at its disposal in pursuit of Global Campaign objectives. The aim of this study was to remedy this: to formulate a definition of quality of care specific to headache which would (1) have relevance to people with headache, (2) apply across the spectrum of headache disorders and the range of health-care settings, (3) extend beyond clinical indicators and (4) maintain its utility across countries, cultures and health-care systems.

## Methodology

Our approach was to identify the specific and measurable elements of quality which, taken together, would ground its definition. This meant that quality indicators would give rise to the definition of quality rather than vice versa. Quality indicators address specific, explicitly defined and measurable elements of practice [14, 16, 17]. Their development is based on evidence, on stakeholders’ views and on consensus [18]. Accordingly we advanced in several steps: a structured review of the literature, a qualitative study comprising stakeholder focus-group discussions, and two international consultations with stakeholder representatives (Fig. 1). The findings from the literature review are published elsewhere [19]. The qualitative study and stakeholder consultations, along with the final product, are described here.

## Qualitative study

Three focus groups were conducted, each involving one type of stakeholder: doctors, nurses and people with headache (the last hereafter referred to as patients). Participants in these groups were selected by convenience sampling, but also purposively: we aimed to include participants who were broadly representative of the types of professionals and patients to whom the indicators might apply. All the patients who participated reported high-frequency headache, and were probably not representative, so their input was supplemented by interviews with two less-severely affected people with headache.

Semi-structured discussion guides were developed from themes emerging from the literature review. These guides were used to inform the group discussions, which nonetheless were allowed to take any relevant direction, with further questions introduced as they progressed. In addition to direct questions on headache-care quality, the doctors’ and nurses’ guides included questions on their roles within and experience of providing headache care. The guide for patients was designed to gain insight into the experience of receiving headache care. All three guides encouraged participants to talk about perceived strengths and weaknesses of headache services and to offer suggestions for improving headache care.

The focus groups and interviews were digitally recorded and professionally transcribed. The transcripts (raw data) were subjected to a thematic analysis by MP and SJ who compared their findings. This means that themes emerged from the data rather than being imposed prior to analysis. The analysis aimed to identify themes that participants considered most important in their relation to quality of care. A comparative approach identified differences in the views and experiences of the various stakeholders.

## Consultations and development of quality indicators

The themes emerging from the literature review and qualitative study provided the basis of a first draft, listing every element that appeared to be part of what each source recognised as quality in headache care. These elements were in multiple domains: diagnosis, treatment, referral for care, outcomes (including quality of life and disability) and satisfaction with care. Education of health-care professionals was regarded as an important contributor to good-quality headache care, both in the literature [20] and by the focus groups. However, we considered it beyond the scope of quality indicators to set out criteria for the education of health professionals. Guidelines for headache education were already being developed [21], and our underlying assumptions were that health-care professionals were trained adequately and that, if they were not and if this had an impact on quality of care, other measures would show it.

The development of quality indicators from this first long list of 160 putative indicators in multiple domains was an iterative process. We reviewed, refined and shortened the list, applying our knowledge and expertise as headache specialists and health services researchers from several countries (Denmark, Germany, Portugal, Sri Lanka, UK and US). We reduced the number of domains by collapsing some: for example, four initial domains of clinical history, clinical examination, investigations and diagnosis were collapsed into a single domain “diagnosis”. Duplications were deleted, as were items considered too specific, not indicators of quality or not relevant across settings. Others were deleted when there was no consensus to include them.

Broader stakeholder consultations were the next step, involving two electronic surveys in which the list of putative indicators was e-mailed to stakeholder representatives in many countries. We first circulated the shorter list of indicators to 33 members of a review panel established previously by *Lifting The Burden* for the development of cross-cultural management aids [22]. They were in 16 countries across all six WHO regions (Africa, the Americas, Europe, Eastern Mediterranean, South East Asia and Western Pacific). We asked whether listed domains and indicators covered all relevant aspects, and whether any were not part of quality. We also asked whether each indicator, in the respondents’ opinions, was “essential for good-quality headache services”; when it was not, we asked whether a headache service “would be a better headache service if it was included as an element of quality”.

We reviewed the outcome of the consultation and further refined the list according to consensus. At this point we compared our domains with the six put forward by the IOM [10]. We then undertook a second and much wider consultation. All members of the International Headache Society, national delegates of the European Headache Federation and representatives of lay organization members of the World Headache Alliance were invited by e-mail to participate, along with a large list of people, in all regions of the world, who had professional or personal interests in headache and had initially been recruited by WHO and *Lifting The Burden* as contributors to their *Atlas of Headache Disorders* [5]. Because most of these mailings necessarily went through these other agencies, the number consulted is not known, but exceeded 1,000 distributed among >100 countries. We asked respondents to rate each indicator on a scale of 0 (not important) to 9 (very important), and analyzed these ratings according to the method used by the Organization for Economic Cooperation and Development (OECD) Health Care Quality Indicators project [23]. In this method, mean ratings of 7–9 indicate support for an indicator, 4–6 ambiguity and 1–3 rejection. By adopting these thresholds, we arrived at the final list of indicators.

The primary aim of the project was to define quality. Once consensus was reached on which indicators to include, within which domains, a definition of quality emerged.

## Results

### Focus groups and interviews

Four doctors participated in the doctors’ focus group, all from the UK: one secondary-care headache specialist, one neurologist and one general practitioner each with a special interest in headache, and one academic general practitioner with a special interest in headache research. Four nurses were in the nurses’ group, three from the UK and one from Denmark. One was a research nurse, whereas the other three were clinical nurses specializing in headache. The patients’ group included five women, aged 31–64 years, with headache disorders. Three described themselves as having chronic daily headache while two reported migraine with attacks at least twice a month. The additional interviews included two women with headaches less than once a month.

Five themes were identified from the focus groups and interviews: (1) headache services, (2) health professionals, (3) patients, (4) financial resources and (5) political agenda and legislation. We describe each of these briefly. The emphasis placed by the three stakeholder groups on each theme was different, and the last two themes were not discussed by the patients. Given the purpose of the study and the interest in defining quality of headache services, the first theme was discussed more extensively within all focus groups and interviews than the other four.

### Headache services

Within this theme, participants described their views of treatment and current headache services, suggesting how they might be improved. The health professionals’ view was that services were driven by individual professionals with an interest, rather than centrally. There was general agreement that headache treatment must be individual to the patient according to diagnosis and temporal variation of the headaches. Health professionals acknowledged that some headaches were difficult to treat and some patients mismanaged, and that inadequate training and lack of appropriate interest in headache were contributors to this. Patients reported that finding the right treatment was often a matter of trial and error, and that their treatment needs changed over time as their headaches changed. Patients described both positive and negative experiences with doctors and, like the professionals, believed not all doctors had sufficient expertise or interest in managing headache disorders.

Doctors called for improvements in access to services (i.e., provision of local services), in individualized care, in simple, basic and fundamental rules of headache service provision across all levels (primary through to tertiary care) and in good referral systems. Nurses and patients emphasized the importance of appropriate referral systems, local care, follow-up and a multidisciplinary team approach. Nurses thought services should focus on “realistic outcomes”, which they defined as those improving quality of life but which, they stressed, included informing patients that, although headache disorders could be treated, they could not be cured. Patients with frequent headaches believed improved services would offer better access to specialists, shorter waiting times, a more holistic approach to headache management and doctors more open to alternative therapies. Patients with less frequent headaches wanted other health professionals, such as community pharmacists, to be more involved in headache care, making effective medications available without the need to consult doctors for repeat prescriptions.

### Health professionals

The second theme embraced the types of health professionals needed in headache care, such as general practitioners, neurologists and specialist nurses, and a multidisciplinary team approach. There was consensus that not all health professionals had the necessary expertise to diagnose and treat headache well. Training of health professionals was considered fundamental, particularly by the doctors, for good-quality headache services. The team approach, involving different types of health professional, was emphasized mostly by the doctors and nurses. Patients attached importance to the doctor–patient relationship, which was not always satisfactory, and this underpinned their perceptions of suboptimal headache care.

### Patients

Health professionals discussed patients’ expectations and their differing needs, again highlighting the importance of individualized treatment. Patients focused on their headache symptoms and their impact, and on their initiatives to achieve better care including the use of health services and alternative therapies. The patients with less frequent headaches seemed more confident about how to manage their headaches and more comfortable relying on medications, believing these to be effective. The patients with more frequent headaches were more reluctant to take medication. Patients described themselves as proactive and self-driven in finding appropriate treatment, and nurses believed that patients should be empowered and taught to self-manage their headaches. Patients thought they received little information about headaches and their management from health professionals.

### Financial resources and political agenda/legislation

These two themes were discussed only by the doctors and nurses. Both were considered important, as no health service could provide high-quality care without adequate funding and appropriate legislation. The health professionals believed interest in headache care could be stimulated and developed successfully only if headache care were on the political agenda. One doctor described headache as the “poor relation” within the wide family of diseases, meaning that, politically, headache received little attention. Both doctors and nurses thought there was insufficient evidence to show which services were cost-effective. They stressed the need for health economic research, as well as regular audits of existing headache services.

## First stakeholder consultation

The first draft list of 160 items in 14 domains (Table 1) was reduced to 50 items in 8 domains. Of the panel of 33 reviewers, 18 responded (11 neurologists, one headache specialist, one GP with a special interest in headache, one nurse, one researcher in headache and three patients or patient representatives), returning completed questionnaires. These provided, between them, a broad opinion base, which led to 23 of the 50 items being rejected: 13 were duplicated or sufficiently addressed by another, four were deemed too general, one was too context-dependent and there was no consensus over five. Despite general agreement that diagnostic indicators were essential, the majority within this category were rejected, being covered by a single indicator requiring universal use of ICHD-II criteria [24] for the diagnosis of headache. One new indicator was added to the “individualized management” domain: this covered waiting time for the first appointment.

**Table 1:** Initial domains of quality

1	Clinical history taking
2	Clinical examination
3	Investigations (such as MRI or CT scan) for headache disorders
4	Diagnosis of headache disorders
5	Medical treatment for headache disorders
6	Consultations and referrals
7	Outcome
8	Education and training of health-care professionals
9	Perceptions of health-care professionals (e.g., satisfaction or interest in headache)
10	Delivery of care
11	Education of patients
12	Patients’ perceptions (e.g., expectations, preferences or understanding of care)
13	Patient satisfaction of care
14	Cost-effectiveness of care

Comparison of the retained domains with those of the IOM revealed considerable overlap, but the IOM had one domain—“safety”—missing from our list. This had not emerged from the review of the literature or the stakeholder consultations. Safety was considered important for quality of headache care, and we added this domain and three indicators of it.

## Second stakeholder consultation

The list e-mailed for the second, wider consultation therefore included 31 putative indicators in nine domains. A total of 157 surveys were returned, two of which were not included in the analysis as they contained no data. Most respondents were headache specialists (*n* = 65, 41.9 %) or neurologists (*n* = 61, 40.6 %). The remainder were other medical doctors, nurses, psychologists, physiotherapists, headache patients or representatives of patient organizations. They came from 45 countries, with the USA (*n* = 32, 20.6 %), Italy (*n* = 23, 14.8 %) and UK (*n* = 11, 7.1 %) most represented. All six WHO regions were represented in the sample: 86 (55.5 %) from Europe, 51 (32.9 %) from the Americas, four (2.6 %) from South East Asia, four (2.6 %) from Africa, three (1.9 %) from Western Pacific and three (1.9 %) from Eastern Mediterranean.

The mean scores were >7 (the threshold for retention) for all indicators except two: “outcome measures are based on economic consequences of headache” (mean 6.3) and “costs of the service are measured as part of a cost-effectiveness policy” (mean 6.6). The former was deleted, but we retained the latter because costs of services are an aspect of health care that cannot realistically be ignored. We deleted one indicator scoring >7 (“treatment plans reflect remediable aggravating factors”) because we believed it was not measurable. We moved one indicator (“patients are not over-investigated”) from the safety domain to the efficiency domain. One additional indicator was added (“patients are asked about the onset of their headaches”). This led to a final set of 30 quality indicators in nine domains (Table 2), all deemed essential and none claiming especial importance.

**Table 2:** The 30 agreed quality indicators for headache care

Domain A: Accurate diagnosis is essential for optimal headache care	
A1	Patients are asked about onset of their headaches
A2	Diagnosis is according to current ICHD criteria
A3	A working diagnosis is made at the first visit
A4	A definitive diagnosis is made at first or subsequent visit
A5	Diagnosis is reviewed during later follow-up
A6	Diaries are used to support or confirm diagnosis
Domain B: Individualized management is essential for optimal headache care	
B1	Waiting-list times for appointments are related to urgency of need
B2	Sufficient time is allocated to each visit for the purpose of good management
B3	Patients are asked about the temporal profile of their headaches
B4	Treatment plans follow evidence-based guidelines, reflecting diagnosis
B5	Treatment plans include psychological approaches to therapy when appropriate
B6	Treatment plans reflect disability assessment
B7	Patients are followed up to ascertain optimal outcome
Domain C: Appropriate referral pathways are essential for optimal headache care	
C1	Referral pathway is available from primary to specialist care
C2	Urgent referral pathway is available when necessary
Domain D: Education of patients about their headaches and their management is essential for optimal headache care	
D1	Patients are given the information they need to understand their headache and its management
D2	Patients are given appropriate reassurance
Domain E: Convenience and comfort are part of optimal headache care	
E1	The service environment is clean and comfortable
E2	The service is welcoming
E3	Waiting times in the clinic are acceptable
Domain F: Achieving patient satisfaction is part of optimal headache care	
F1	Patients are satisfied with their management
Domain G: Optimal headache care is efficient and equitable	
G1	Procedures are followed to ensure resources are not wasted
G2	Patients are not over-investigated
G3	Costs of the service are measured as part of a cost-effectiveness policy
G4	There is equal access to headache services for all who need it
Domain H: Outcome assessment is essential in optimal headache care	
H1	Outcome measures are based on self-reported symptom burden (headache frequency, duration and intensity)
H2	Outcome measures are based on self-reported disability burden
H3	Outcome measures are based on self-reported quality of life
Domain I: Optimal headache care is safe	
I1	Patients are not over-treated
I2	Systems are in place to be aware of serious adverse events

From these indicators the following multidimensional definition of *quality of headache care* was formulated:

Good-quality headache care achieves accurate diagnosis and individualized management, has appropriate referral pathways, educates patients about their headaches and their management, is convenient and comfortable, satisfies patients, is efficient and equitable, assesses outcomes and is safe.

## Discussion

Our starting belief was that good-quality care could not be achieved if it was not known what it was. Initiatives to improve care would serve little purpose if (a) it was unknown in what direction(s) improvement lay, and (b) improvement could not be recognized. Although general definitions of quality of care had been proposed, there were none for the essential specifics of headache care, with its individualized requirements. Suggested quality indicators for headache [12, 13] had limited application, and would not serve *Lifting The Burden*’s purpose of improving care, or of implementing care where none existed, in countries throughout the world. A new set of quality indicators was needed, along with a definition of quality, both grounded on the consensus views of key stakeholders.

Quality is clearly multidimensional. The process of defining it requires that the domains in which quality resides are *all* identified. If all are agreed to be part of quality, they need not be prioritized within a definition. This is not to say they may not be ranked in importance at the point of service-implementation: indeed, there must be some prioritization because resource limitations induce competition between the aspects of quality. An obvious example is in the allocation of time: what is given to one patient in achieving individualized care (the IOM domain of patient/family-centredness) is at the opportunity cost of treating others (jeopardizing the IOM domains of timeliness, equity and, possibly, efficiency). Prioritization, however, is a matter for local determination according to local resources, views, culture and expectations. What we have done is to create a template of quality, available as a guide. It is also a basis for standard-setting, if that is required, although this was not our main purpose.

Our nine domains of quality (diagnosis; individualized treatment; referral; education of patients; convenience and comfort; patient satisfaction; efficiency and equity; outcome; safety) reflect but are not identical to, and go beyond, Donabedian’s seven pillars (efficacy; effectiveness; efficiency; optimality; acceptability; legitimacy; equity) [9] and the IOM’s six domains of quality (safety; timeliness; effectiveness; efficiency; equity; patient/family-centredness) [10]. They are headache specific, which is what we set out to achieve. They also go beyond the domains previously identified for quality of headache care: symptoms, examinations and medications in the US [12], and diagnosis, referral and treatment in the UK [13].

The definition of quality that we built on these nine domains is disaggregated, based on multiple elements which collectively constitute quality [11]. Disaggregated definitions allow greater specificity than generic definitions, and have more relevance in their application to services within a particular field such as headache.

The development was driven by collaboration between experts, in headache on the one hand and in health-services research on the other. This was a strength of the study. The focus groups were small, and drawn almost entirely from the UK; while this might be seen as a limitation, focus groups are no more than a starting point, along with the literature review, for identifying themes. Other methodologies build upon this. Focus groups are not in themselves intended to be, and realistically cannot be, highly representative. In the next stage we received and took account of inputs from larger groups much more representative of the same three key stakeholders in headache services (doctors, nurses and patients), drawn from 45 countries in all world regions. The sample of responders was not perfect, and this too was a limitation, but a very broad base of opinion from >100 countries was consulted, with the opportunity to say something if it was considered important. We believe that this grounding was sound, and that relevance is assured both cross-culturally and cross-contextually, although the truth of this belief needs to be tested empirically.

The stakeholder groups we chose not to consult were health-service managers and politicians (the latter being representatives of the general public). Both (or all three) might have views on the meaning of “quality”, but we did not feel these views should underpin the definition of quality. The job of managers is not to decide what services people want, or what shape or size they should be, but to implement the services that evidence shows people do want. The job of politicians is to allocate resources that managers need, but it is also to decide on local priorities as described above, taking account of popular will. In this, they should take an objective view of quality, again informed by evidence, and, like the judiciary, they have no legitimate basis for influencing the evidence. A politician might, with sound reason, claim that an aspect of quality was locally unachievable: equity, for example, because the resources were not there. It would still be an essential part of quality, and if it were not achieved it should remain apparent that quality was deficient in this respect.

Interestingly, “safety” emerged late in the process, and only through comparison with the IOM’s generic description of quality of care [10]. No stakeholder introduced it. The reason, probably, is that it was taken for granted.

Within the nine domains we specify 30 quality indicators that may be utilized to assess a headache service with the purpose of guiding its improvement. Quality indicators should be measurable and relate to relevant elements of health care, and they should provide an understanding of the quality of a health-care system [25] by signaling the existence of deficiencies. Their use is retrospective, and they generate review criteria by which to assess services or care [16]. Our next step, currently in planning, is to evaluate the 30 quality indicators empirically, establishing their suitability and feasibility of use. We will do this in clinical settings.

## Conclusions

We have defined quality of headache care. We conclude that quality in this context is multidimensional, residing in nine domains of equal importance, and is assessed by a set of 30 quality indicators. These indicators are different from previously developed indicators in that they were developed with international input from representatives of various stakeholder groups, including patients, are specific to headache (without being specific to any one type of headache), and address a wider range of dimensions of quality. They are intended to guide headache-service improvements and/or implementation in Global Campaign initiatives conducted by *Lifting The Burden* worldwide.
